# Dominant-feature Identification in Data from Gaussian Processes Applied to Finnish Forest Inventory Records

**DOI:** 10.1007/s13253-025-00706-5

**Published:** 2025-07-26

**Authors:** Roman Flury, Tuomas Aakala, Leena Ruha, Timo Kuuluvainen, Reinhard Furrer

**Affiliations:** 1 Forest Resources and Management, Swiss Federal Institute for Forest, Snow and Landscape Research WSL, Birmensdorf, Switzerland; 2 Department of Mathematics, University of Zurich, Zurich, Switzerland; 3 Faculty of Science and Forestry, University of Eastern Finland, Joensuu, Finland; 4 Natural Resources Institute Finland (Luke), Oulu, Finland; 5 Department of Forest Sciences, University of Helsinki, Helsinki, Finland; 6 Department of Mathematical Modeling and Machine Learning, University of Zurich, Zurich, Switzerland

**Keywords:** Basal area, Cold forest, Covariance analysis, Georeferenced data, Scales, Spatial patches

## Abstract

Conventional geostatistical methods often assume a single process across spatial scales, potentially masking scale-dependent patterns that originate from distinct underlying processes. Particularly, nearby locations exhibit similar values and thereby form connected structures—features—that vary across scales. While scale-space analysis aims to disentangle such overlapping structures and reveal scale-dependent features, there is no method available to detect statistically credible features in geostatistical data. Here, we introduce a scale-space decomposition method for identifying features in Gaussian process-modeled geostatistical data, which also enables the estimation of scale-dependent effects of predictor variables. Features are defined as statistically credible, scale-dependent structures identified by significant deviations from zero between differences of successive smooths of the data. To demonstrate these capabilities, we applied the approach to Finnish forest inventory data from the 1920s. We identified two essential spatial scales in basal area of common tree species: plot-to-plot variation and regional scale. Our scale-dependent analysis reveals that edaphic factors consistently influence all species across scales, while anthropogenic drivers show contrasting scale-specific effects: slash-and-burn agriculture negatively affects spruce at both scales but shows opposite effects on birch at different scales. These insights advance the understanding of historical forest ecology and demonstrate the utility of our approach.

## Introduction

According to the first law of geography (Tobler 1970), everything is related to everything, but nearby things are more so than distant things. This principle implies that nearby locations in spatial data often exhibit more similar values than those farther apart, such as similar canopy heights of neighboring trees, comparable soil moisture in adjacent plots or consistent land-cover types across a region. These local similarities form connected spatial structures that can vary across different scales. For example, the canopy heights of a small community of trees regenerating under similar local conditions may display features at a local scale, while the canopy heights of an entire forest, influenced by broad climatic trends, might form features at a larger scale. Local-scale features can reflect fine-grained processes such as sunlight or seedbed availability among neighboring trees, whereas large-scale features are shaped by landscape-level factors such as climate gradients or soil properties. As a consequence, we observe different features depending on the spatial scale. As these scale-dependent features likely result from different underlying processes influenced by distinct factors, understanding these processes requires identifying the relevant spatial scales and the dominant features present at each. This makes it possible to analyze the driving factors behind each process separately, advancing our understanding of how these systems and their structures are shaped. Analogous to Holmström et al. (2011), we define features as those structures that are statistically credible and scale-dependent, identified by significant deviations from zero between differences of successive smooths of the data. Smoothing suppresses noise and reveals features on increasingly larger spatial scales. By taking the differences between successive smooths, these features are further separated, enabling the disentangling of overlapping spatial patterns. The entire process of detecting these scales, identifying their statistically credible features, and evaluating their properties is referred to as *dominant-feature identification* (Flury et al. 2021).

To address these multi-scale spatial processes computationally, various statistical modeling approaches have been developed that utilize multi-scale representations for computational efficiency when analyzing spatially distributed data. For instance, multi-scale Gaussian Markov random fields (GMRFs) (Nychka et al. 2015) or multiresolution approximations of spatial covariance functions (Katzfuss 2017) have been developed to facilitate scalable inference for large datasets. While these approaches exploit multiple scales for numerical tractability, they do not necessarily assume that the data originates from distinct underlying processes at different scales. The idea of multiresolution approximation was extended by Paige et al. (2022) to involve Bayesian inference by integrated nested Laplace approximation (INLA) (Lindgren et al. 2011; Bakka et al. 2018). Zammit-Mangion and Rougier (2020) proposed to model multi-scale data with non-stationary underlying processes using stacked processes. These processes are approximated with GMRFs to exploit the conditional dependence structure of the latent variables. More recently, Zhang and Katzfuss (2022) made use of the so-called Vecchia approximation for multi-scale processes, assuming that the scales are known a priori. A related body of work in multi-fidelity modeling addresses similar ideas from a different perspective, aiming to combine information from sources of varying resolution or accuracy—referred to as low- and high-fidelity data—to achieve efficient and robust predictions. Notably, Kennedy and O’Hagan (2000) introduced a Bayesian framework for fusing low- and high-fidelity models, and recent extensions such as warped multi-fidelity Gaussian processes allow the modeling of skewed environmental data (Colombo et al. 2025). While these approaches primarily focus on computational efficiency, parallel developments in scale-space analysis have emphasized the explicit identification of scale-dependent features in spatial data.

Building on these approaches, statistical scale-space multiresolution analysis (Holmström et al. 2011; Holmström and Pasanen 2017; Pasanen et al. 2018) identifies individual scales in spatial data by systematically decomposing spatial structures. Originating in signal processing (Witkin 1983; Lindeberg 1994), this approach was later adapted in statistical scale-space analysis (Chaudhuri and Marron 1999) and extended to include Bayesian significant zero crossings of derivatives (Erästö and Holmström 2005). These approaches and their implementations have been optimized but remain limited to complete and regularly gridded data (pixel-based images). Flury et al. (2021) and Flury and Furrer (2019) extended these ideas to spatial data assigned to a graph structure, including missing values, irregularly gridded and areal data using GMRFs. Crucially, they introduced the concept of *dominant-feature identification*, a scale-space analysis for detecting statistically scale-dependent dominant features—features that are prominent at a given scale—and for assessing their attributes, such as their spatial extent. Scale-space analysis have since been successfully applied in various studies (Lehmann et al. 2017; Aakala et al. 2018; Kulha et al. 2019).

In gridded or areal data, each value represents a larger area, whereas in geostatistical data (often also termed georeferenced data), a value corresponds to a specific point at a fixed location (Cressie 1993). A key contribution of the work presented here is the extension of the *dominant-feature identification* to geostatistical data. In this scenario, the values at the specific locations are treated as a realization of a spatial process, which allows modeling correlations between locations based on the distance between locations rather than simply neighboring structures as is the case with a GMRF. Section 2 describes the mathematical concepts and assumptions to fill this gap in the scale-space literature.

Beyond identifying features at different scales, understanding how external predictor variables relate to spatial patterns is often of scientific interest, especially when these patterns vary across spatial scales. In our context, once dominant features have been identified at different scales, it becomes relevant to assess whether and how predictors (such as environmental or site-specific variables) are associated with these features. Crucially, this enables a scale-specific interpretation of covariate effects, revealing nuanced associations that may otherwise be masked and missed in aggregated analyses (Pasanen and Holmström 2017). While scale-dependent correlation between two time series has been explored (e.g., in Pasanen and Holmström, 2017; Kulha et al, 2019), a framework for scale-dependent multiple regression modeling in spatial data, conditional on identified features, has been missing so far.

We assume that features are scale-dependent in spatial data. Simply applying regression models therefore risks masking these scale-dependent relationships, as the large-scale structure can dominate and obscure the small-scale dependencies. In our method, we first extract features at different scales to accurately capture scale-specific dependency structures. In the subsequent steps, we apply scale-dependent regression analysis using a set of predictor variables to identify their associations with the dominant features and assess the statistical significance of such associations. This novel aspect, introduced here to geostatistical data, is adaptable also for existing *dominant-feature identification* approaches for other spatial data and thus closes another essential gap. Assessing whether a spatial scale is statistically significant can be accomplished, for example, through permutation test against the null hypothesis that there are no spatial features, as proposed by Kulha et al. (2019). We introduce here a cross-validation approach that allows assessing the quality of the scales and the following estimates.

In order to establish the power of the outlined feature identification method for geostatistical data (in Sect. 2), we applied it to historical forest inventory data collected in Finland in the 1920s (see Sect. 3). This dataset includes detailed observations of basal area for Finland’s three most common tree species: pine (*Pinus sylvestris*), spruce (*Picea abies*), birch (*Betula pendula* and *B. pubescens*), and other deciduous trees. These data, observed before the onset of modern forestry, carry insight into the natural and anthropogenic processes that determined how forests developed at the time. Specifically, the scales at which different features emerge were still unknown before this analysis, and provide further insight into the past. These results are especially valuable for understanding the historical development of forest resources, biodiversity, and carbon stocks. The introduced method and the analysis of the Finnish forest inventory data are implemented in the statistical software R (R Development Core 2023) and are openly available in the repository associated with this manuscript (Section A of the Supplementary Material).

## Dominant-feature Identification Method

In this section, we provide the details of the *dominant-feature identification* method for geostatistical data. We consider the situation where $\boldsymbol{y}$ is the variable of interest and is observed data at $n \in \mathbb {N}^+$ distinct locations $\boldsymbol{s} \in \mathcal {D} \subseteq \mathbb {R}^2$, and we assume that the observed data comprises a realization $\boldsymbol{x} = (x_1, x_2, \ldots , x_n)^\top$ of a spatial process $\{ X(\boldsymbol{s}): \boldsymbol{s} \in \mathcal {D} \subseteq \mathbb {R}^2 \}$ and an independent noise component. We write $\boldsymbol{X}$ as the finite-dimensional representation of the process $X(\boldsymbol{s})$ observed at the *n* locations. Thus, we express the model as

1 $$\begin{aligned} \boldsymbol{Y} = \boldsymbol{X} + \boldsymbol{\varepsilon }, \end{aligned}$$

assuming $\boldsymbol{y}$ is a realization of the random vector $\boldsymbol{Y}$, and $\boldsymbol{\varepsilon } \sim \mathcal {N}_n \left( \boldsymbol{0}, \sigma ^2_{\boldsymbol{\varepsilon }} \boldsymbol{I}_n \right)$. We further assume $X(\boldsymbol{s})$ is a zero-mean Gaussian process whose finite-dimensional covariance matrix $\boldsymbol{\Sigma }$ is defined through a covariance function $\text {COV}(X(\boldsymbol{s}_i), X(\boldsymbol{s}_j))=\text {cov}(||\cdot ||; \boldsymbol{\theta })$ for all locations $\boldsymbol{s}_i,\boldsymbol{s}_j \in \mathcal {D}$ and additional covariance parameters $\boldsymbol{\theta }$ (Cressie 1993). Note that the representation of $\boldsymbol{Y}$ can be extended with a location-dependent mean function. Throughout this paper, we use the isotropic Matérn covariance function (other covariance functions could be used instead, e.g., Flury and Furrer, 2022). The covariance parameters $\boldsymbol{\theta }$ therefore consist of σ>0, the marginal standard deviation (partial sill); ν>0, the smoothness parameter; and ρ>0, the effective range parameter that controls the distance of spatial correlation. With the parameterization after Lindgren et al. (2011), ρ is defined as the distance at which the spatial correlation is approximately 0.13. Then,

2 $$\begin{aligned} \text {cov}(||\boldsymbol{s}_i - \boldsymbol{s}_j||; \boldsymbol{\theta }) = \frac{\sigma ^2}{2^{\nu - 1} \Gamma (\nu )} (\sqrt{8\nu }||\boldsymbol{s}_i - \boldsymbol{s}_j||/\rho )^{\nu } \text {K}_{\nu } (\sqrt{8\nu }||\boldsymbol{s}_i - \boldsymbol{s}_j||/\rho ). \end{aligned}$$

Here, Γ is the Gamma function and $\text {K}_\nu$ is the modified Bessel function of the second kind of order ν.

We use the likelihood function of the Gaussian process to estimate the covariance function parameters. For large data sets, low-rank approximations could be applied to optimize the likelihood function more efficiently. Also, the outlined dominant-feature identification are not restricted to data following Gaussian processes. Other processes are applicable, for example data following a Poisson process (Flury and Furrer 2019; Agarwal et al. 2002) or using a suitable link function (Miller et al. 2020).

### Field Reconstruction

In Model (1), we assume that the observed data $\boldsymbol{y}$ is a noisy realization of a composition of $\boldsymbol{X}$ and some observational or measurement noise $\boldsymbol{\varepsilon }$. To estimate $\boldsymbol{X}$, we propose, for example, so-called conditional sampling, which enables reconstruction of the field along with an explicit quantification of uncertainty. Here, we are using the fact that $\boldsymbol{X}$ and $\boldsymbol{\varepsilon }$ both follow a zero-mean Gaussian distribution, which implies that

3 $$\begin{aligned} \boldsymbol{Y} \sim \mathcal {N}_n\left( \boldsymbol{0}, \boldsymbol{\Sigma } + \sigma ^2_{\boldsymbol{\varepsilon }}\boldsymbol{I}_n \right) . \end{aligned}$$

Furthermore, it holds that

4 $$\begin{aligned} \begin{pmatrix}\boldsymbol{X}\\ \boldsymbol{Y} \end{pmatrix} \sim \mathcal {N}_{2n} \left( \boldsymbol{0}, \left( \begin{array}{lllll} \boldsymbol{\Sigma } \quad & & & & \boldsymbol{\Sigma }\\ \boldsymbol{\Sigma } \quad & & & & \boldsymbol{\Sigma }+\sigma ^2_{\boldsymbol{\varepsilon }}\boldsymbol{I}_n \end{array}\right) \right) . \end{aligned}$$

Moreover, as $\boldsymbol{\varepsilon }$ is assumed to be independent random noise, $\boldsymbol{X}$ and $\boldsymbol{\varepsilon }$ are independent, and the cross-covariance submatrices are equivalent to $\boldsymbol{\Sigma }$. The conditional distribution $\boldsymbol{X}|\boldsymbol{Y} = \boldsymbol{y} \sim \mathcal {N}_n \left( \boldsymbol{\Sigma }(\boldsymbol{\Sigma } + \sigma ^2_{\boldsymbol{\varepsilon }}\boldsymbol{I}_n)^{-1}\boldsymbol{y}, \boldsymbol{\Sigma } - \boldsymbol{\Sigma }(\boldsymbol{\Sigma } + \sigma ^2_{\boldsymbol{\varepsilon }}\boldsymbol{I}_n)^{-1}\boldsymbol{\Sigma } \right)$ then follows again a multivariate Gaussian distribution. We can therefore calculate the mean (i.e., $\text {E}[\boldsymbol{X}|\boldsymbol{Y}=\boldsymbol{y}]$) directly. However, we propose sampling from the conditional distribution, which enables a credibility analysis of the *dominant-feature identification* approach.

For efficient sampling, particularly when computing the inverse of Σ becomes computationally expensive, we can draw samples from conditional distribution using the following steps (Rasmussen and Williams 2006):

1. Sample realizations $(\boldsymbol{x}_1^\top , \boldsymbol{y}_1^\top )^\top$ of $(\boldsymbol{X}^\top , \boldsymbol{Y}^\top )^\top$ using the density function and covariance matrix according to Eq. (4).
2. Transform the realizations from $(\boldsymbol{X}^\top , \boldsymbol{Y}^\top )^\top$ to $\boldsymbol{X}|\boldsymbol{Y} = \boldsymbol{y}$ by performing $\boldsymbol{x}_1 + \boldsymbol{\Sigma }(\boldsymbol{\Sigma } + \sigma ^2_{\boldsymbol{\varepsilon }}\boldsymbol{I}_n)^{-1}(\boldsymbol{y} - \boldsymbol{y}_1)$.

We set the parameters of the covariance function defined in Eq. (2) using a maximum likelihood (ML) approach according to the Distribution (3) or based on characteristics in the data. The variances of the noise $\sigma ^2_{\boldsymbol{\varepsilon }}$ are set using the observed data $\boldsymbol{y}$. As a result, only one additional linear system based on $(\boldsymbol{\Sigma } + \sigma ^2_{\boldsymbol{\varepsilon }}\boldsymbol{I}_n)$ needs to be solved (Furrer et al. 2024).

Approximate conditional sampling approaches can be applied if necessary, for example as described in Bailey et al. (2021) or, for observations on the sphere, in Emery et al. (2019). As an alternative to an ML approach, if prior knowledge is available about the support of the sample space, a Bayesian hierarchical model (Gelfand 2012) can be used instead of conditional sampling. In this case, $\text {E}[\boldsymbol{X}|\boldsymbol{Y}=\boldsymbol{y}]$ denotes the posterior sample mean.

### Decomposition

In classical scale-space analysis, as described by Holmström et al. (2011), the data $\boldsymbol{x}$ (as defined in Model (1)) are smoothed using a roughness penalty smoother applied over increasing smoothing scales. The data are then decomposed as the sum of the differences of these smooths at successive scales. In general, a roughness penalty smoother is defined as $\boldsymbol{S}_{\lambda } = (\boldsymbol{I}_n + \lambda \boldsymbol{Q})^{-1}$, where λ is the smoothing scale and $\boldsymbol{Q}$ is an arbitrary spatial weight matrix. As we introduced a spatial process in Model (1), we express a smoother in terms of the correlation matrix $\boldsymbol{R}$ such that the entries of $\sigma ^2\boldsymbol{R}$ are given by Eq. (2), with

5 $$\begin{aligned} \boldsymbol{\widetilde{S}}_{\lambda } = \sigma ^2\boldsymbol{R} (\sigma ^2\boldsymbol{R} + \lambda \boldsymbol{I}_n)^{-1}. \end{aligned}$$

Let here $\boldsymbol{Q}$ represent the precision matrix of the spatial process $X(\boldsymbol{s})$, then the smoother $(\boldsymbol{I}_n + \lambda \boldsymbol{Q})^{-1}= \boldsymbol{Q}^{-1}( \boldsymbol{Q}^{-1} + \lambda \boldsymbol{I}_n)^{-1} = \sigma ^2\boldsymbol{R} (\sigma ^2\boldsymbol{R} + \lambda \boldsymbol{I}_n)^{-1}$ is conceptually equivalent to the roughness penalty smoother. Analogous to the general definition of the penalty smoother, it holds that $\lim _{\lambda \rightarrow 0} \boldsymbol{\widetilde{S}}_{\lambda }\boldsymbol{x} = \boldsymbol{x}$, and $\lim _{\lambda \rightarrow \infty } \boldsymbol{\widetilde{S}}_{\lambda }\boldsymbol{x} = \overline{\boldsymbol{x}}$, the overall mean of $\boldsymbol{x}$, which is by assumption $\overline{\boldsymbol{x}} = \boldsymbol{0}$.

The correlation matrix $\boldsymbol{R}$ in the definition of $\boldsymbol{\widetilde{S}}_{\lambda }$ and $\sigma ^2$ are based on the Matérn covariance function, whose parameters affect $\boldsymbol{\widetilde{S}}_{\lambda }$. We propose to choose the smoothness parameter ν of the Matérn covariance function such that it represents the smoothness of $\boldsymbol{x}$ adequately, and estimate the partial sill $\sigma ^2$ and effective range ρ parameter based on $\boldsymbol{x}$.

To decompose $\boldsymbol{x}$, we consider a sequence of smoothing scales $0 = \lambda _1< \lambda _2< \ldots < \lambda _{L} = \infty$ to construct the smoother $\boldsymbol{\widetilde{S}}_{\lambda }$ and apply it to $\boldsymbol{x}$. Thereby, $\boldsymbol{\widetilde{S}}_{0}\boldsymbol{x} = \boldsymbol{x}$ and $\boldsymbol{\widetilde{S}}_{\infty }\boldsymbol{x} = \boldsymbol{0}$ by assumption of the overall mean of $\boldsymbol{x}$. Then, $\boldsymbol{x}$ can be represented as $\boldsymbol{x} = \boldsymbol{\widetilde{S}}_{0}\boldsymbol{x} - \boldsymbol{\widetilde{S}}_{\lambda _2}\boldsymbol{x} + \boldsymbol{\widetilde{S}}_{\lambda _2}\boldsymbol{x} - \ldots + \boldsymbol{\widetilde{S}}_{\lambda _{L-1}}\boldsymbol{x} - \boldsymbol{\widetilde{S}}_{\infty }\boldsymbol{x}$. The differences $\boldsymbol{z}_{\ell } = (\boldsymbol{\widetilde{S}}_{\lambda _{\ell }} - \boldsymbol{\widetilde{S}}_{\lambda _{\ell +1}} )\boldsymbol{x}$, for $\ell = 1, \ldots , L-1$ are referred to as details, and $\boldsymbol{x}$ can be expressed as a sum of details, $\boldsymbol{x} = \sum _{\ell = 1}^{L-1} \boldsymbol{z}_{\ell }$. In practice, this decomposition is calculated for each sample draw from Sect. 2.1, and the details are summarized by their sample mean. In the following, we simplify notation and denote the summarized details as $\boldsymbol{z}_{\ell }$.

### Scale selection

In order to select sensible smoothing scales, we follow Pasanen et al. (2013), who introduced the idea of scale derivatives that can be adapted for the spatial smoother $\boldsymbol{\widetilde{S}}_{\lambda }\boldsymbol{x}$, analogously defined by $\widetilde{\boldsymbol{D}}_{\lambda }\boldsymbol{x} = \frac{\partial \boldsymbol{\widetilde{S}}_{\lambda }}{\partial \log \lambda }\boldsymbol{x}$. As the degree of smoothing increases, the difference between successive values for λ has to become larger and more significant to have a noticeable effect on the smoothing, which is why the scale derivative λ is on a logarithmic scale. The scale derivative can be expressed in terms of the smoothing correlation matrix $\boldsymbol{R}$ as

6 $$\begin{aligned} \widetilde{\boldsymbol{D}}_{\lambda } \boldsymbol{x} = -\lambda \sigma ^2\boldsymbol{R} (\sigma ^2\boldsymbol{R} + \lambda \textbf{I}_n)^{-1} (\sigma ^2\boldsymbol{R} + \lambda \textbf{I}_n)^{-1}\boldsymbol{x}. \end{aligned}$$

This scale derivative shows the change of the field dependent on the smoothing scale λ. We choose λ corresponding to the local minima of the scale derivative with respect to either the Euclidean norm or the maximum norm (Königsberger 2003). In this manner, the difference between the corresponding smoothers captures all scale-dependent dominant features related to the maximum between respective minima (Pasanen et al. 2013). Flury et al. (2021) showed that the maximum norm is more sensitive and can detect local extremes that may arise from anisotropic or non-stationary spatial processes.

We provide a simulation study in Supplementary Material B to demonstrate the effect of potentially mis-specifying the effective range or smoothness parameters, for the calculation of $\boldsymbol{R}$, on the smoothing scale selection. Here, we assume that $\sigma ^2$ is estimated correctly as it is a scaling factor in the Matérn covariance function (see Eq. 2) and keep it constant in the simulation. The results show that the minima of the scale derivative shift only marginally as the effective range and smoothness parameter values increase. Therefore, the effect on the decomposition is minimal.

### Credibility Analysis

To credibly recognize the dominant scale-dependent features, we calculate the probability of a value at a given location being either positively or negatively different from zero based on the individual samples from the field reconstruction (Sect. 2.1) and the derived smoothing scales. As the credibility analysis of dominant-feature identification inherently depends on the choice of the selected smoothing scales $\lambda _{\ell }$, a careful selection of these scales is crucial for robust conclusions (see Sect. 2.6). Here, we use so-called pointwise (PW) credibility maps to recognize credible dominant features. For the PW map of the ℓth detail $\boldsymbol{z}_{\ell }$, considered as a realization of the random vector $\boldsymbol{Z}_{\ell }$, each location $\boldsymbol{s}_i \in \mathcal {D}$ is allocated to subsets $I^\text {high}=\{ i: \text {P}(\boldsymbol{Z}_{\ell , \boldsymbol{s}_i} > 0\mid \boldsymbol{y}) \ge \alpha \}$, $I^\text {low}=\{ i: \text {P}(\boldsymbol{Z}_{\ell , \boldsymbol{s}_i} < 0\mid \boldsymbol{y}) \ge \alpha \}$ or to $I^\text {null}=\{1,\dots ,n\}\setminus \left( I^\text {high} \cup I^\text {low}\right)$, in which the detail $\boldsymbol{z}_{\ell ,s}$ credibly differs from zero locationwise with respect to the credibility level α, typically $\alpha = 95\%$. In PW maps, probabilities are calculated independently for each location. Other credibility maps, such as *highest pointwise probabilities* or *simultaneous credible intervals*, increase the coherence of credible locations (Erästö and Holmström 2005; Holmström et al. 2011). Furthermore, Bolin and Lindgren (2015) proposes to estimate *excursions* and *contour uncertainty regions* for latent Gaussian models based on a parametric family for the excursion sets combined with posterior samples.

### Feature Attributes

In order to complete the dominant-feature identification method for geostatistical data, we assess the characteristics of each detail. We estimate spatial properties, i.e., the covariance function parameters and, as a novel aspect, the scale-dependent linear fixed effects of additional predictor variables on the same scale as the respective detail. We denote the whole set of covariance function parameters and linear effects of a detail as feature attributes. We can model the details $\boldsymbol{z}_{\ell }$ for $\ell =1,\, \ldots , \, L-1$, representing the observed spatial data $\boldsymbol{y}$ at different scales, with a spatial component and linear effects of *k* predictor variables. Therefore, we use Gaussian processes $V_{\ell }(\boldsymbol{s})$ at the $n \in \mathbb {N}$ locations $\boldsymbol{s} \in \mathcal {D}$ and $T_{\ell } \sim \mathcal {N}_n(\boldsymbol{0}, \tau _{\ell }^2\textbf{I}_n)$ (where $\tau _{\ell }^2 \ge 0$ corresponds to the spatial nugget effect) and we model the observed spatial data $\boldsymbol{y}$ as

7 $$\begin{aligned} \boldsymbol{y}&= \boldsymbol{x} + \boldsymbol{\varepsilon } = \sum _{\ell = 1}^{L-1} \boldsymbol{z}_{\ell } + \boldsymbol{\varepsilon } = \sum ^{L-1}_{\ell = 1} \Bigl [\boldsymbol{w}_{\ell }(\boldsymbol{s})^\top \boldsymbol{\beta }_{\ell } + \boldsymbol{V}_{\ell }(\boldsymbol{s}) + \boldsymbol{T}_{\ell } \Bigr ] + \boldsymbol{\varepsilon }\nonumber \\&=\sum ^{L-1}_{\ell = 1} \Bigl [ \boldsymbol{w}_{\ell }(\boldsymbol{s})^\top \boldsymbol{\beta }_{\ell }\Bigr ] + \boldsymbol{\varepsilon } + \sum ^{L-1}_{\ell = 1} \Bigl [ \boldsymbol{V}_{\ell }(\boldsymbol{s}) + \boldsymbol{T}_{\ell }\Bigr ]. \end{aligned}$$

We define $\boldsymbol{v}(\boldsymbol{s}) = \boldsymbol{\varepsilon } + \sum ^{L-1}_{\ell =1} \boldsymbol{V}_{\ell }(\boldsymbol{s}) + \boldsymbol{T}_{\ell }$ and $\boldsymbol{V} = [v(\boldsymbol{s}_1), \ldots , v(\boldsymbol{s}_n)]^\top$ with

$$\boldsymbol{V} \sim \mathcal {N}_n(\boldsymbol{0}, \boldsymbol{\Sigma }) \quad \text {and} \quad \boldsymbol{\Sigma } = \sum ^{L-1}_{\ell =1} \boldsymbol{\Sigma }_{\ell , \text {spatial}}.$$

Thereby, $\boldsymbol{w}_{\ell }(\boldsymbol{s})$ is the vector of scale-dependent predictors at location $\boldsymbol{s}$. The associated design matrix $\boldsymbol{W}\hspace{-3.0pt}_{\ell } \in \mathbb {R}^{n \times k}$ contains the rows $\boldsymbol{w}_{\ell }(\boldsymbol{s}_i)^\top$ for i=1,…,n, and $\boldsymbol{\beta }_{\ell } \in \mathbb {R}^k$ the linear coefficients. We propose constructing the scale-dependent design matrix by decomposing the predictor variables with the derived smoothing scales for $\boldsymbol{x}$. In particular, for predictors containing robust small- and large-scale features, this potentially leads to essential insights that cannot be obtained using un-decomposed predictors (Pasanen and Holmström 2017).

To estimate the feature attributes for each detail $\boldsymbol{z}_{\ell }$, an ML approach can be used for each addend of the sums of Eq. (7) (Flury and Furrer 2022). When the data size is moderate, for instance the Hessian matrix can be calculated to derive Wald confidence intervals for each estimate (Held and Sabanés Bové 2013). Otherwise, if the main interest is in the associations between the observed data $\boldsymbol{y}$ and scale-dependent predictor variables, then Eq. (7) could be solved as a single system.

### Overfitting

Overfitting is a common problem with statistical models, which can arise when identifying dominant features in spatial data. Choosing too many scales leads to details containing remarkably similar scale-dependent features. Moreover, while estimating feature attributes, overfitting may arise when the model describes the random error in a detail rather than the associations to predictor variables. We propose spatial cross-validation to address these issues. The locations $\boldsymbol{s} \in \mathcal {D}$ are divided into different subareas of approximately equal shape and size (for example, into rectangular-shaped blocks). We then assign these blocks to *k* different training sets, each leaving approximately 1/*k*th of the data out, identify dominant features for each training separately, and analyze whether the scale derivative and the selected scales are similar compared to the scales based on the entire data. Local extrema may affect the analysis if the training set suggests remarkably different scales. This could also indicate that the assumption of isotropy is inappropriate.

Overfitting can be quantified by comparing predictions from Model (7) using, on the one hand, the estimates based on the training sets and, on the other hand, the respective test-set locations from the sum of all details $\boldsymbol{z}_{\ell }$. Classical measures and scores, such as the root-mean-squared error (RMSE) and the continuous ranked probability score (CRPS) (Gneiting et al. 2007), can be calculated.

Additional, but herein not used approaches to tackle overfitting are to use the generalized likelihood ratio hypothesis test, to test whether the partial sill $\sigma ^2_{\ell }$ for each detail process is significantly different from zero, or to use a regularized likelihood approach, broadly in the spirit of Dambon et al. (2021).

### Method Summary

The key steps of the outlined *dominant-feature identification* can be summarized into the following steps:

1. Draw samples of $\boldsymbol{X}|\boldsymbol{Y}=\boldsymbol{y}$, separating noise from observations $\boldsymbol{y}$.
2. Determine smoothing scales used for decomposing $\boldsymbol{x}$ with scale derivatives, based on the sample mean of $\boldsymbol{X}|\boldsymbol{Y}=\boldsymbol{y}$.
3. Calculate details based on individual sample draws of $\boldsymbol{X}|\boldsymbol{Y}=\boldsymbol{y}$ and summarize by their sample mean, simultaneously deriving PW probability maps.
4. Assess feature attributes for each detail $\boldsymbol{z}_{\ell }$ by estimating associations to predictor variables at the same scales and spatial effects by ML.
5. Assess overfitting by repeating the previous steps for *k* subsets.

## Applying Dominant-feature Identification to Finnish Forest Inventory Data

Having established the methodological framework for dominant-feature identification in geostatistical data, we now present its application to Finnish forest inventory data from the 1920s. This application serves both to validate our approach and to address substantive questions in forest ecology that require understanding scale-dependent spatial processes. The following analysis illustrates how the methodological steps outlined in Sect. 2 can reveal ecological insights that would be masked by conventional single-scale approaches.

### Motivation

Natural processes and human activities change forest structures and species compositions. These changes have generally increased over the past centuries, with increasing human population, variety of forest uses, and later the rise of the forest industries. While a variety of data sources, such as remote sensing and forest inventory data, give us a sound understanding of how forests are changing in managed and protected areas in intensively managed regions such as Fennoscandia (Tomppo et al. 2008), the interplay between natural forest development and the influence of human actions prior to modern forestry is much less well known. In particular, the spatial features across large regions and their different scales are unknown. Identifying these features and scales would improve our understanding of the factors behind these features. Analyzing such scientific questions is possible for the Finnish forests by considering available nationwide forest inventories completed by field crews in the last century.

Finland’s first systematic nationwide forest inventory (NFI1) was conducted between 1921 and 1924 (Ilvessalo 1927). The aim was to provide a reliable statistical description of the forest and tree stocks. Following the digitalization of these data, recent studies have complemented the picture that emerged from the original data, with a focus on the development of the number of large and old trees and as well as on tree-size distributions in different regions (Henttonen et al. 2019, 2020). Aakala et al. (2023) recently developed interpolated maps based on a subjective selection of the degree of smoothing to demonstrate larger-scale variability in several forest characteristics. However, whether the regional division or the subjectively chosen smoothers correspond to actual scales in the data remained unknown. By applying the outlined identification of dominant features to the NFI1 data, we can identify these different scales and compare the scale-dependent features among different tree species, taking into account the influence of edaphic and anthropogenic variables.

### Ecological Context

To understand the ecological context of our analysis, we note that Finland is situated roughly between latitudes 60 and 70 North and longitudes 21 and 33 East. Climatic conditions correlate strongly with changes in latitude. For example, the length of the growing season reflects this correlation, which in the forested part of the country ranges from 180 days in Helsinki in southern Finland to only 120 days in Sodankylä in the north. The majority of the Finnish landscape is forested, with boreal forests currently covering more than 70% of the country. The most common tree species in Finland are Scots pine (*Pinus sylvestris*), Norway spruce (*Picea abies*), and birch (*Betula pendula* and *B. pubescens*). Other native deciduous trees, such as aspen and alder, complete Finland’s tree stock. A common attribute assessed for tree stocks is the basal area (BA), which is a structural stand characteristic typically used to describe forest stand density and a proxy for timber volume. BA is measured as the amount of area occupied by tree stems per unit area, typically expressed as $\text {m}^2/\text {ha}$. BA is influenced by site productivity (soil, topography, climate), forest age, species compositions, and disturbance history (either natural disturbances or logging). When developing naturally, the species dominance is mainly influenced by site productivity and the occurrence of fires. Forests on productive sites (both well- and poorly-drained) typically develop into spruce-dominated stands after an initial dominance of deciduous trees. Conversely, on low-productive and dry sites, pine tends to dominate. On intermediate sites, the absence of fires increases dominance of spruce, which is a fire-intolerant species. Conversely, fire occurrence tends to favor pines on these sites.

Given this ecological background, we hypothesize regarding the predictor variables of BA data that differences at small scales in BAs of different species are driven by differences in edaphic conditions (site-type), while at larger scales, after controlling for climatic influence, this variation reflects geographical variation in how people used the forests. First, we expect the commonness of slash-and-burn agriculture to lead to a decline in spruce and an increase in deciduous trees. This is because the more productive spruce-dominated sites are primary areas for slash-and-burn agriculture and because spruce is intolerant of the fire that occasionally escaped from the slash-and-burn areas to the adjacent forests. The areas typically regenerate with deciduous trees following the abandonment of cultivated areas. Second, forest grazing tends to favor other deciduous trees, including species such as alder, which is unpalatable to cattle. Third, population density reflects the pressure for household consumption of wood, especially for fuel, but also for construction and other material uses. We therefore expect especially pine, as the preferred wood material for construction, to show a negative relationship with human influence (Dalheimer and Aakala 2024).

### NFI1 Data

In order to test the hypothesis outlined above, we consider the NFI1 data and data from other historical sources described in the following. For this foremost forest inventory, field crews measured forest on inventory lines from southwest to northeast across Finland, with a 26 km distance between most lines (for details, see  Ilvessalo 1927; Henttonen et al, 2019). The cumulative length of these lines is 13’348 km, without considering areas covered by water. At these inventory lines, sample plots of size $10\,\text {m} \times 50\,\text {m}$ were considered to assess land-use and site characteristics. In the original outline, the distance between plots was 2 km. However, sample plot locations did not have exact coordinates, but their locations were recently determined in Aakala et al. (2023). An assessment based on the comparison between railroad maps of the time and railroad crossings recorded by the inventory crews in the field showed that 90% of the sample plots were within 8 km of their “true” location.

**Fig. 1:**
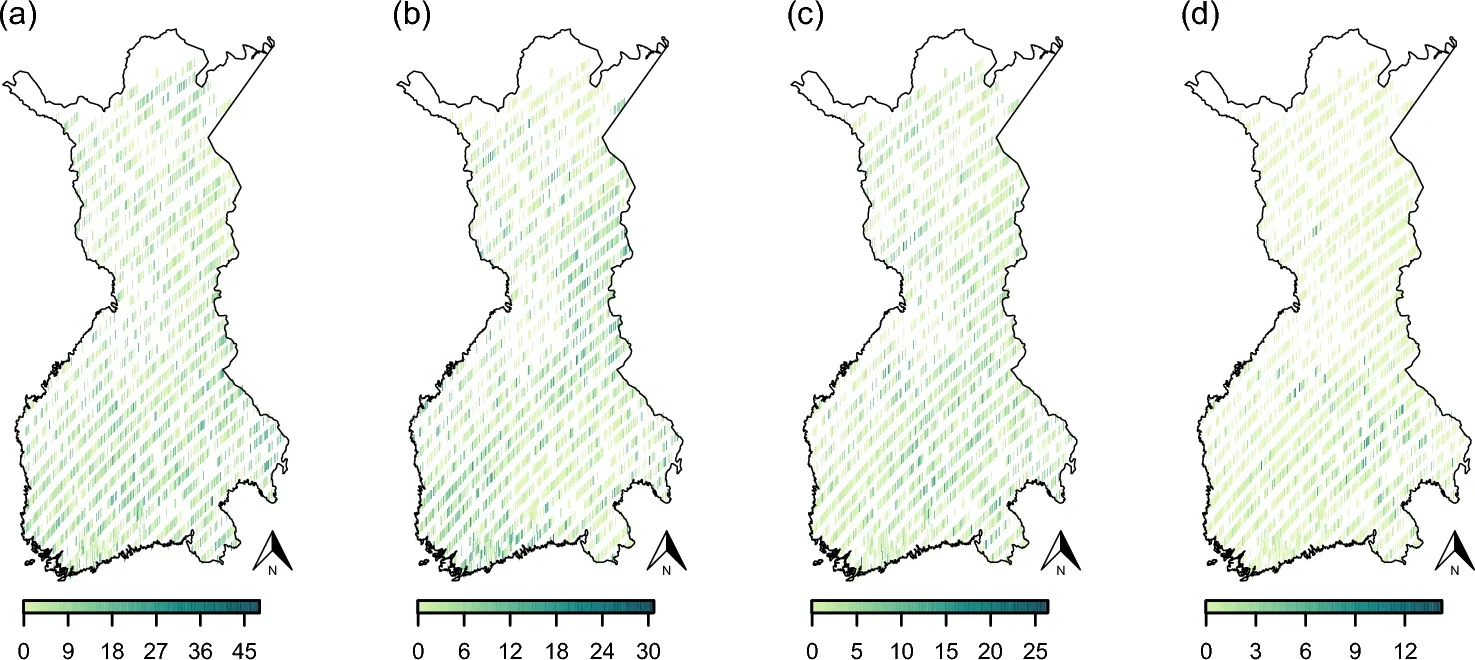
BA based on NFI1: for **a** pine; **b** spruce; **c** birch; **d** other deciduous trees. To enhance visibility, we used a vertical bar as the plot symbol to visualize the values at NFI plots

The NFI1 field crews measured the diameter at breast height (dbh), 1.3 m above ground, of each tree for all $10\,\text {m} \times 50\,\text {m}$ sampling plots. Trees were classified into 2 cm classes so that trees larger than 4 cm were consistently recorded in all plots (4–6 cm, 6–8 cm, and so on). We computed the plot-level BA, based on the dbh measures, by transforming the dbh to the area of a circle; that is, $\text {BA} = \pi \cdot (\text {dbh}/2)^2$. In the NFI1 data, the dbh measures are separately available for pine, spruce, birch, alder, aspen, and other broadleaf trees, where the last three are summarized as “other deciduous trees” (see panels (a) to (d) of Fig. 1). In total, the data set contained 3’065 BA estimates on forests on mineral soil. A visual inspection of the BAs in Fig. 1 implies high and uniform occurrence of pine throughout Finland compared to all other tree species. The second-highest values are evident in the BA of spruce; however, these appear to be less evenly distributed, and in the southeastern part of the country, there is a very low occurrence of spruce. Based on the BAs of birch, a subordinate occurrence of birch seems to be a fixed component of Finnish forests, with some exceptions where birch are more dominant in forest stands. In particular, birch may dominate forests in the north, close to treeline. Other deciduous trees were abundant only in the southern half of Finland.

**Fig. 2:**
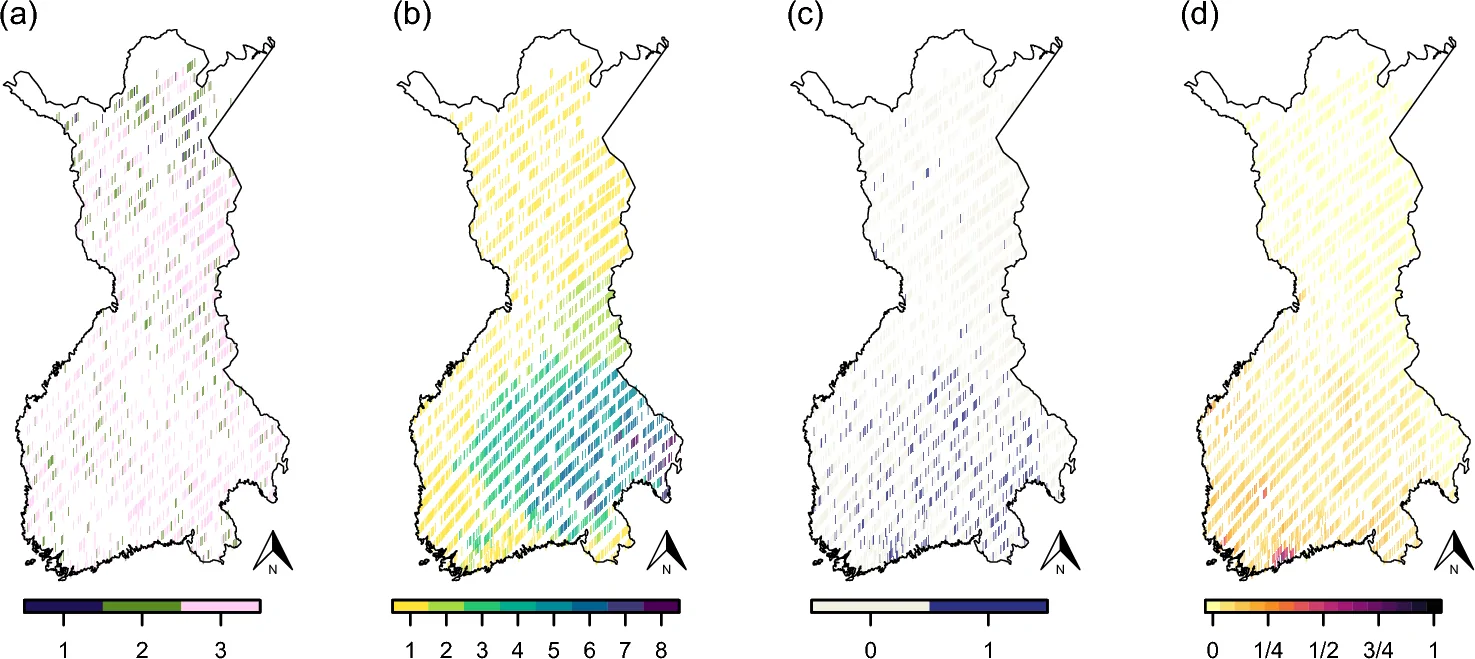
Edaphic and anthropogenic variables: **a** site-type, where the categories *xeric* (1), *sub-xeric* (2), *mesic* (3), correspond to the assigned levels; **b** slash-and-burn agriculture intensity levels; **c** no-grazing or grazing; **d** population density (min-max normalized). To enhance visibility, we used a vertical bar as the plot symbol to visualize the values at NFI plots

During NFI1 sampling, site type was determined for each plot based on a visual assessment. The corresponding productivity classes cover this edaphic influence on the forest stands. We grouped the detailed classification into three broader classes *xeric*, *sub-xeric* and *mesic*, see panel (a) of Figs. 2 and Ilvessalo (1927). Originally, the Finnish equivalent classes from barren to herb-rich were assessed: *karukko*, *kuiva*, *kuivahko*, *tuore* and *lehtomainen*. However, *karukko* and *kuiva* as well as *tuore* and *lehtomainen* were combined with xeric and mesic, respectively, given that there were only a few observations. The most dry-barren and dry sites were found in the very northern part of Finland.

We considered the following three anthropogenic variables to account for human influence on the forests. First, the prevalence of slash-and-burn agriculture, a method of cultivation in which the trees in a forest area are burned and cleared for several years of cultivation, followed by a few decades of rest to let the forest recover. The intensity of this practice at the parish level in 1913 in Finland is shown in panel (b) of Fig. 2; the data are from Heikinheimo (1915). The more intensively this practice was used over these years, the higher the level. In the past century, this practice was used most commonly in the southeast of Finland, where the occurrence of spruce is very low and other deciduous trees are most abundant. Second, the binary variable ‘grazed’ describes whether the respective plot was used for forest grazing, see panel (c) of Fig. 2. Often, cattle were let into forests to graze, which were left to recover from slash-and-burn agriculture cultivation independent of the intensity of this practice. In addition, we consider the population density in 1925 of Finland, see panel (c) of Fig. 2. The continuous values for each plot location are based on an inverse-distance weighted interpolation of population density (Aakala et al. 2023), from the digitalization of a settlement map provided in Witting (1928). The figure shows that Finland is more populated in the south, and the larger cities are concentrated in the southwest.

### Dominant-feature Identification Steps

**Fig. 3:**
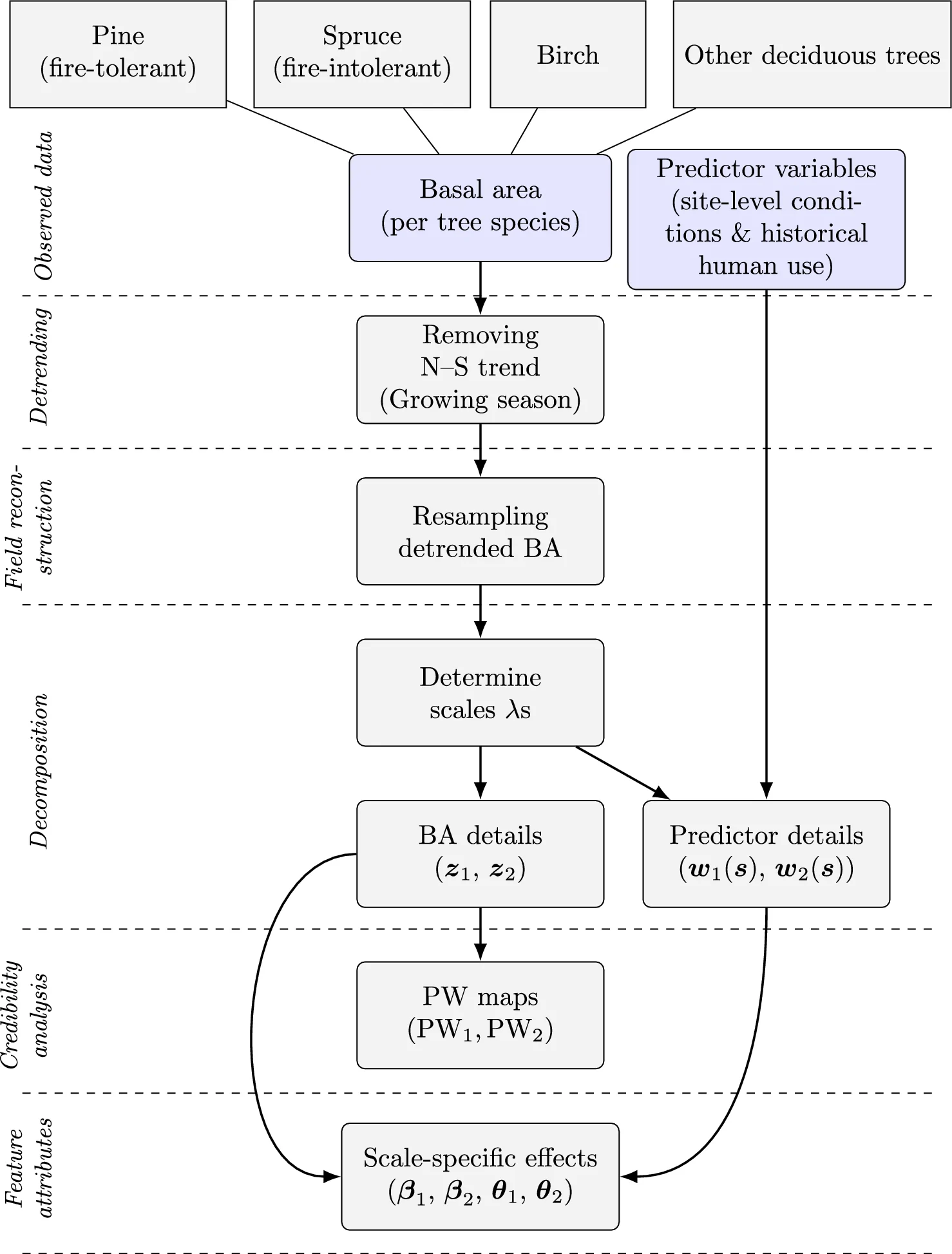
Conceptual workflow illustrating the analysis of basal area (BA) per tree species. Labels on the left indicate the main stages of the analysis pipeline

We now describe the application of the dominant-feature identification procedure summarized in Sect. 2 to the NFI1 basal area data. This application demonstrates how each methodological component—from field reconstruction through feature attribute estimation—works with geostatistical data in practice. Figure 3 provides a graphical overview of the whole analytical workflow, illustrating how each step contributes to identifying and interpreting dominant spatial features at multiple scales. The computational steps were implemented in the statistical software R (R Development Core 2023), and the code openly available in the repository associated with this manuscript (Section A of the Supplementary Material). All steps were equivalently applied to BA of pine, spruce, birch, and other deciduous trees (other). These four datasets included 3’065 data points each, which determined the dimensions of the respective covariance matrix structures. The sizes of the corresponding computational objects were moderate. We applied spatial cross-validation to ensure that the scales and final estimates are not a result of overfitting. Therefore, we divided blockwise (each block measuring size 26 km×26 km) the area of interest into five subsets of approximately 80% of the data and repeated the described steps for each subset.

First, we preprocessed the BA data by removing the natural linear trend in North–South direction as the growth rates of the trees depend on their latitude coordinate, with a decline along these. Omitting the detrend step would imply an additional scale and detail in the decomposition. Therefore, to improve separability and considering that the trend based on the growth rates is not the focus of the outlined hypothesis, we removed this linear trend. Subsequently, we continued with the standardized residuals of these detrended BAs, which are assumed to follow a zero-mean Gaussian distribution. Furthermore, we assumed that the data is a realization of an isotropic spatial process and standardized it such that the mean is zero and the standard deviation is one. Next, we made use of the conditional sampling approach to remove observational and measurement noise $\boldsymbol{\varepsilon }$ from the observed data $\boldsymbol{y}$ according to Model (1). We drew 1’000 random samples and approximate $\boldsymbol{x}$ with the mean of these samples.

**Fig. 4:**
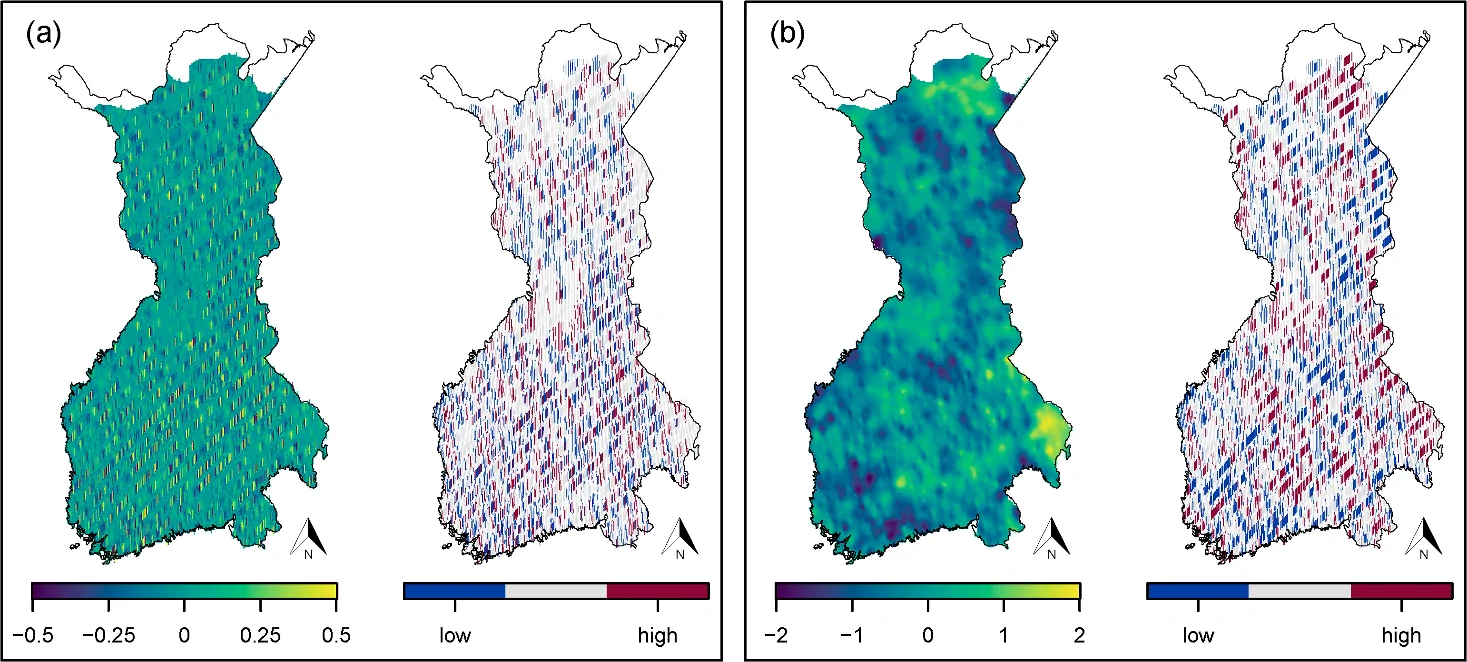
Interpolated scale-space decomposition of pine BA: (a) pine $\boldsymbol{z}_1$ and PW$_1$; (b) pine $\boldsymbol{z}_2$ and PW$_2$. To enhance visibility, we used a vertical bar as the plot symbol for pine $\boldsymbol{z}_1$ and the PW maps. The area in white is above the tree line

To select the scales, we were using the scale derivative defined in Eq. (6), thereby calculating the smoothing correlation matrix according to a Matérn covariance function (Eq. (2)). We set the parameters of this function such that the effective range ρ corresponds to 26 km, ensuring that multiple inventory lines are considered to model the smoothing correlation matrix, and we chose ν=0.5, such that the noisy spatial structure is appropriately reflected. The smoothing correlation matrix was computed based on great-circle distances between all plot locations. Henceforth, we could select the smoothing scales according to the minimas of the maximum norm of the scale derivative. In all four BA data sets, we are able to identify one minimum leading to two details. The scales are (standard deviation of cross-validation results in parentheses) $\lambda _{\text {pine}} = 1,\, (1.16)$, $\lambda _{\text {spruce}} = 0.47,\, (0.45)$, $\lambda _{\text {birch}} = 1.73,\, (0.44)$, and $\lambda _{\text {other}} = 0.27,\, (0.08)$. See Fig. 8 of the Supplementary Material for a complete overview of the calculated scale derivatives.

In the next step, we calculated the decomposition of the BA data, based on the selected smoothing scales, the smoothing correlation matrix and the individual sampling draws. We obtained two respective details for the specific BA data of each species, summarized in their conditional sample means,  pine/spruce/birch/other $\boldsymbol{z}_1$ and $\boldsymbol{z}_2$. We used PW probability maps based on the individual sampling draws from the sampling step to visualize dominant features more clearly. Figure 4 shows both details and PW maps for pine BA and respective figures in Section C of the Supplementary Material show the decomposition for BA data of spruce, birch, and other trees. The respective detail and PW maps were interpolated to the whole of Finland, where a weighted *k*-nearest neighbors approach was used to complete the predictor variables (Hechenbichler and Schliep 2004).

We assessed dominant scale-dependent feature attributes by simultaneously estimating the spatial correlation and the effects of the described edaphic and anthropogenic drivers. Therefore, we used for the respective two details (Model (7)), where the scale-dependent design matrices $\boldsymbol{W}\hspace{-3.0pt}_1$ and $\boldsymbol{W}\hspace{-3.0pt}_2$ are constructed with the decomposed driver variables, $\boldsymbol{\beta }_1$ and $\boldsymbol{\beta }_2$ are the vectors of corresponding scale-dependent linear coefficients, and $V_{1}(s)$, $V_{2}(s)$ are the underlying zero-mean isotropic Gaussian processes, characterized with a Matérn covariance function. As we also decomposed, on the same smoothing scales, the ordered categorical variables site type, slash-and-burn intensity, and grazed to construct the two design matrices, it was possible to assess their scale-dependent effect on the respective details. Their smoothed-effect behavior became similar to a continuous variable and showed the impact of the increasingly ordered factors. The coefficients and covariance function parameters were estimated separately in a joint ML approach for each detail. As the number of data points was not excessively large, we could compute the Hessian matrix while optimizing the ML and construct Wald confidence intervals for each estimate.

### Results

**Fig. 5:**
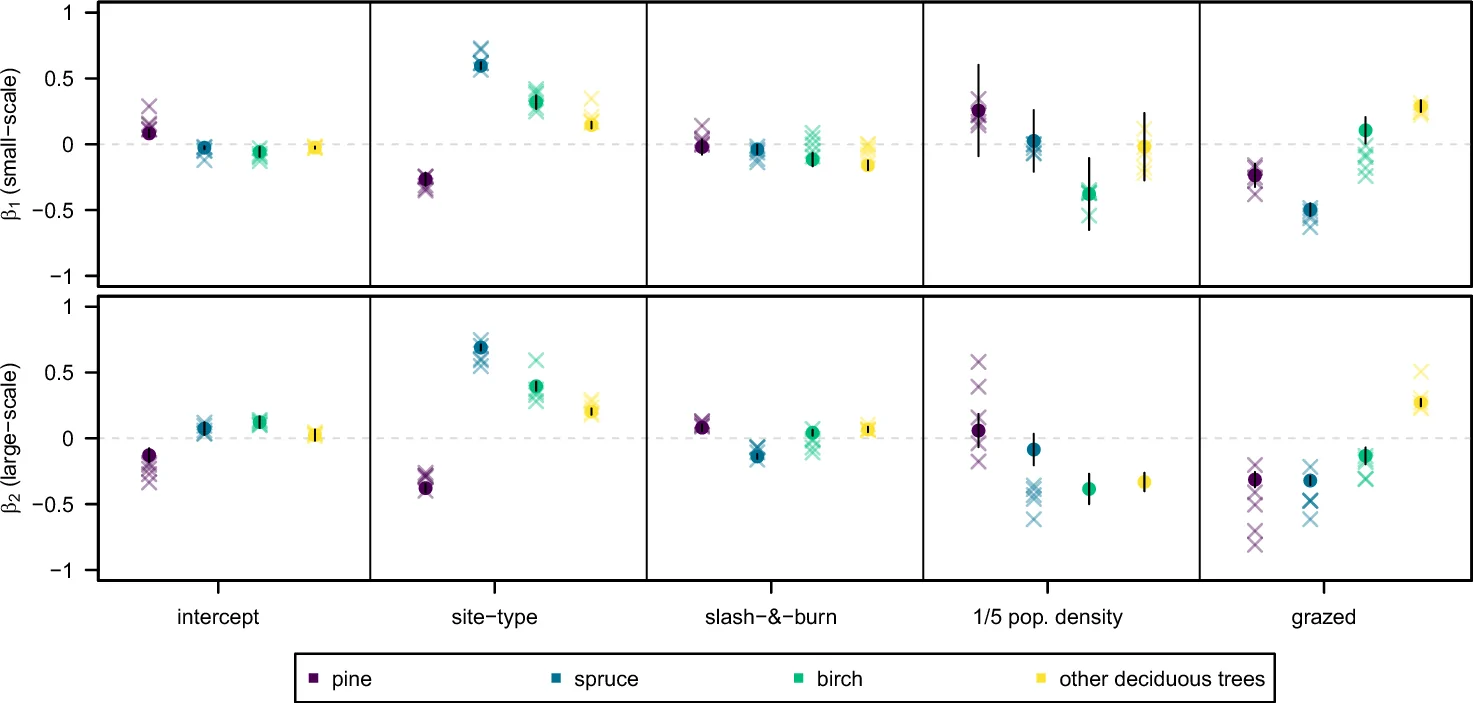
ML estimates of the coefficients of the linear effects of BA detail models at different scales: the upper panel is for the $\boldsymbol{z}_1$- and the lower panel for the $\boldsymbol{z}_2$-models. In each sub-panel, estimates based on the entire data are visualized by dots, cross-validation estimates with crosses, and 95-% Wald confidence intervals with black lines. The respective estimates of population density are scaled by 1/5 for visualization

**Fig. 6:**
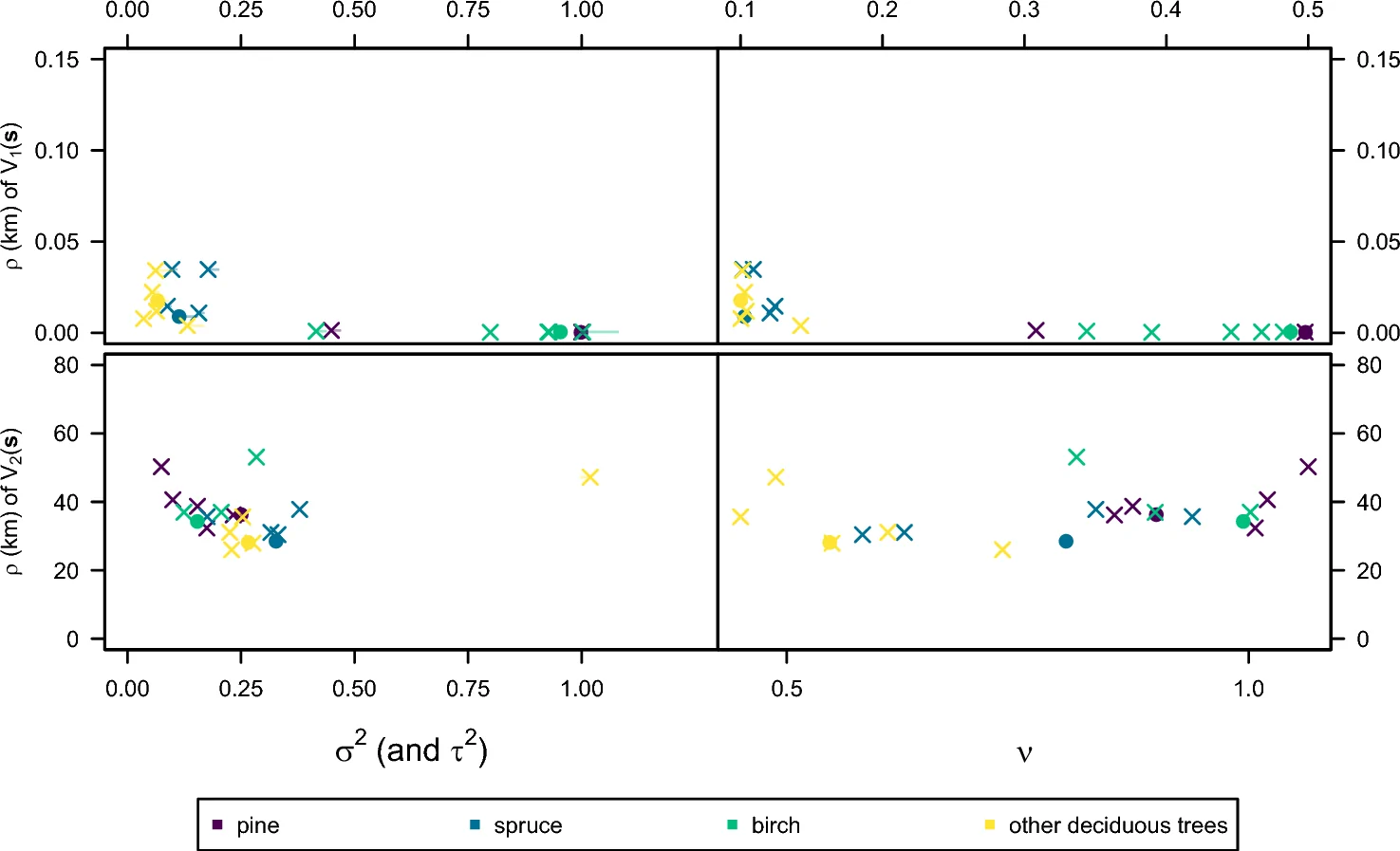
ML estimates of the spatial effects of BA detail models: the upper panel is for the $\boldsymbol{z}_1$- and the lower panel for the $\boldsymbol{z}_2$-models. Estimates based on the entire data are visualized by dots, cross-validation estimates with crosses and nugget effect with horizontal lines

The dominant-feature identification procedure revealed two distinct spatial scales in the Finnish forest data, confirming our methodological approach while providing new ecological insights. We present these results following the analytical sequence established in Sect. 2: first examining the identified scales and their spatial characteristics, then analyzing the scale-dependent effects of environmental and anthropogenic variables (see Fig. 3). This interpretation includes detail and PW maps, as well as the estimates of the linear and the spatial effects. The resulting scale-dependent linear effects according to Model (7) for pine/spruce/birch/other $\boldsymbol{z}_1$ and $\boldsymbol{z}_2$, including cross-validation estimates, are summarized in Fig. 5. The estimates of the spatial components of these models are separately visualized in Fig. 6. For validation purposes, we compare our findings to those of Aakala et al. (2023), who employed boosted regression trees to investigate the influence of different variables from the same dataset, using expert-selected spatial scales.

**Fig. 9:**
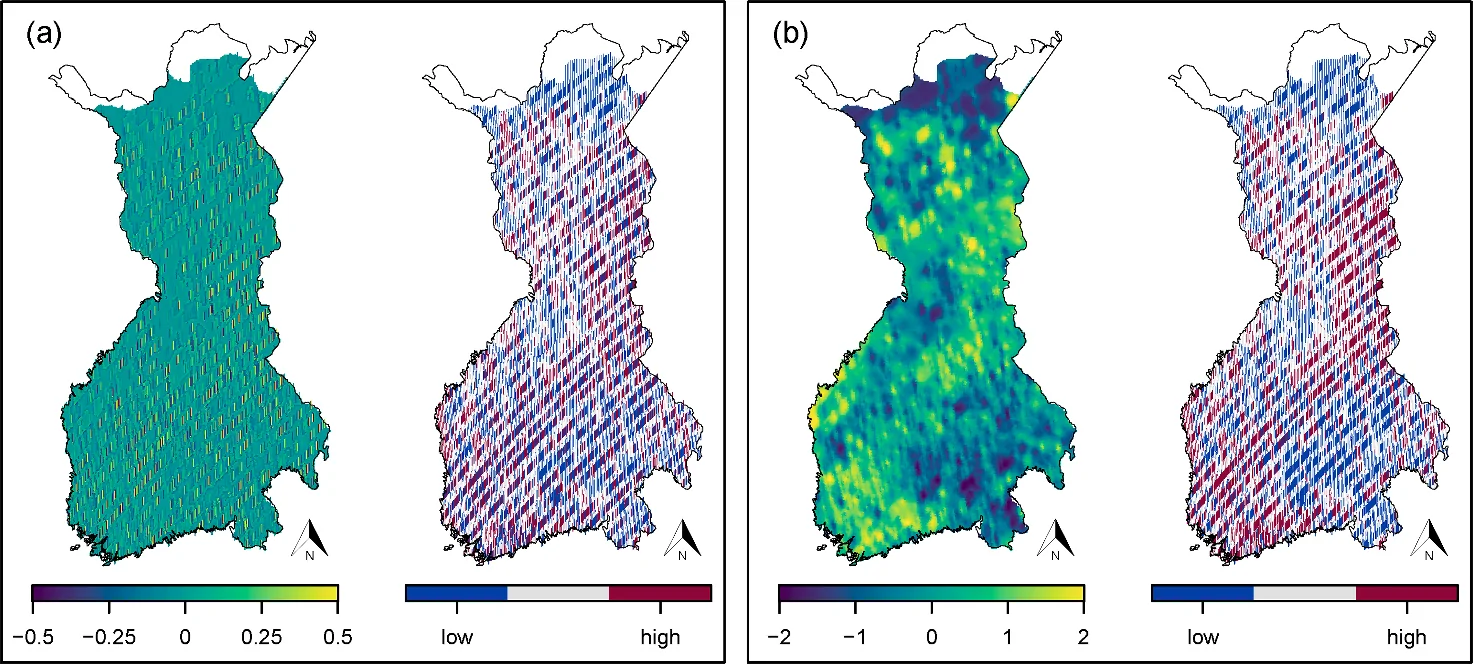
Scale-space decomposition of pine BA: **a** spruce $\boldsymbol{z}_1$ and spruce PW$_1$; **b** spruce $\boldsymbol{z}_2$ and spruce PW$_2$. The area in white is above the tree line

**Fig. 10:**
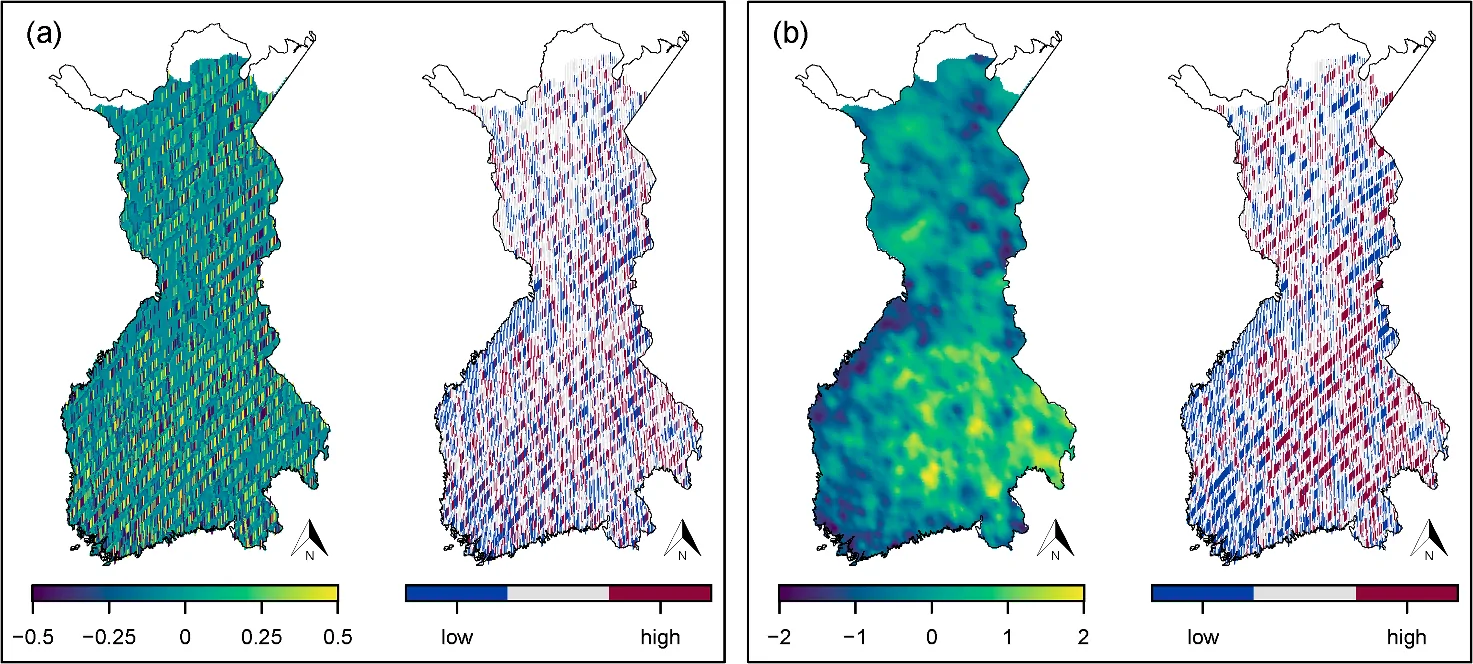
Scale-space decomposition of birch BA: **a** birch $\boldsymbol{z}_1$ and birch PW$_1$; **b** birch $\boldsymbol{z}_2$ and birch PW$_2$. The area in white is above the tree line

**Fig. 11:**
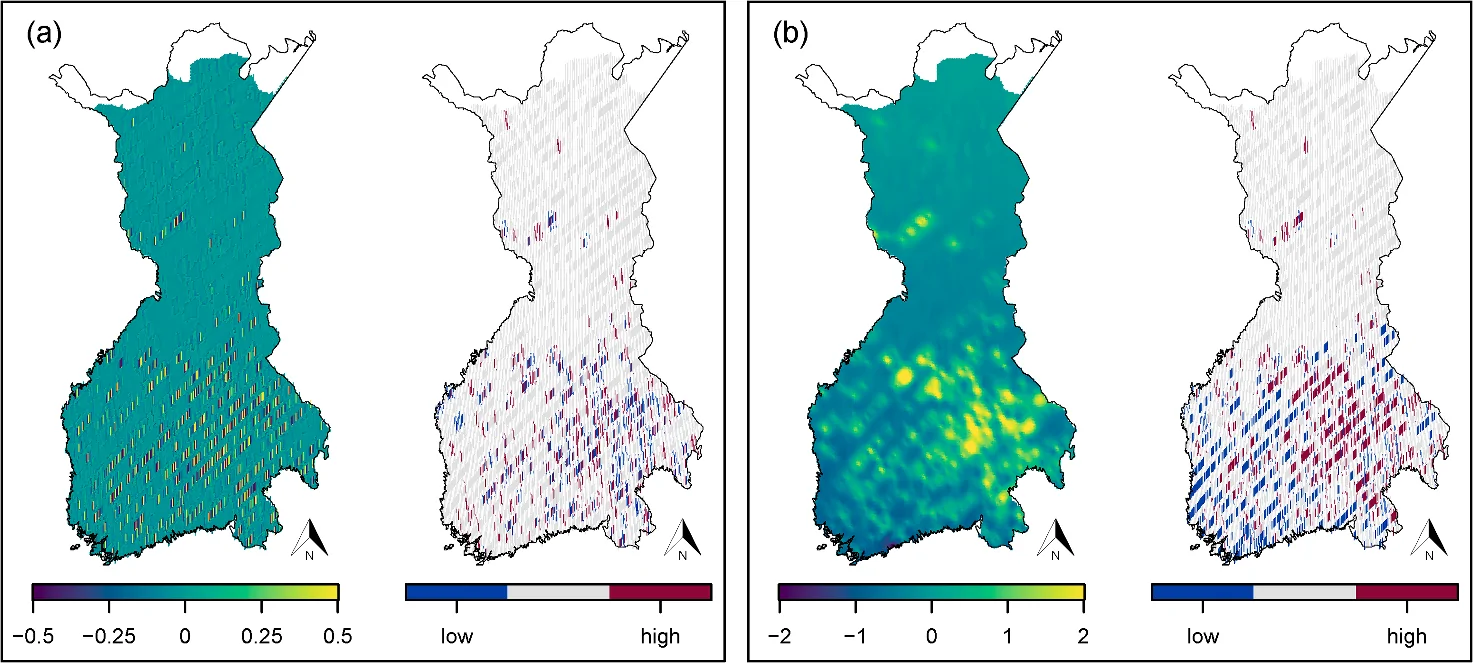
Scale-space decomposition of other BA: **a** other $\boldsymbol{z}_1$ and other PW$_1$; **b** other $\boldsymbol{z}_2$ and other PW$_2$. The area in white is above the tree line

Examining the spatial structure of our results, when comparing overall the details $\boldsymbol{z}_1$ and $\boldsymbol{z}_2$ with the scale-space decomposition (Fig. 4 and Supplementary Material Figs. 9, 10 and 11), we observed that the small-scale details ($\boldsymbol{z}_1$’s) mainly contain plot-to-plot variation. The large-scale details show regional scale-dependent dominant features for all species, and spruce BA seems to have larger features than any of the other tree species.

Turning to the covariate effects, we assessed scale-dependent linear effects of the four variables visualized in Fig. 2 on the scale-dependent BA details (Fig. 5). The intercept, the mean value of the decomposed BAs is for all tree species close to zero.

The site-type effects show the expected ecological demeanor, consistently on $\boldsymbol{z}_1$ and $\boldsymbol{z}_2$. Pine BAs are negatively associated with increasing richer site types, which is consistent with pine being more abundant on poor sites. Spruce, birch, and other deciduous trees that typically dominate more fertile sites show the opposite relationship to pine. That is, the richer the site type, the higher the BAs. These effects are consistent for coniferous trees on small and large scales.

Examining slash-and-burn effects, pine BAs are on $\boldsymbol{z}_2$ positively associated with increasing intensity in such agriculture. However, on $\boldsymbol{z}_1$, there is no significant effect. By contrast, spruce BAs are consistently negatively related to this variable on both small and large scales, in alignment with the hypothesis that this practice was detrimental for spruce. Birch BAs are negatively influenced on $\boldsymbol{z_1}$ from this form of cultivation. However, on $\boldsymbol{z}_2$, the effect on birch BAs becomes positive. The positive association of birch BAs can be explained by the fact that mountain birches are highly abundant in the north of Finland and were not exposed to slash-and-burn agriculture in this region. Moreover, birch usually reappears after a quicker recovery phase than spruce, which explains the different effect on $\boldsymbol{z}_2$ compared to spruce. On $\boldsymbol{z_1}$ of other deciduous trees, BAs are negatively associated with this practice. However, on the large scale, this association becomes positive, as hypothesized. These results are in agreement with the findings of Aakala et al. (2023), who also report spatially heterogeneous impacts of slash-and-burn agriculture on species composition, particularly highlighting negative effects on spruce and more resilient or positive responses of birch in northern regions. Our decomposition further supports this interpretation by disentangling these effects by spatial scale.

In terms of population density effects, birch BAs are consistently negatively associated with population density, and other deciduous trees show no significant effect on $\boldsymbol{z}_1$ but a strongly negative association on $\boldsymbol{z}_2$. Pine and spruce BA show no significant association with population density on $\boldsymbol{z}_1$ and $\boldsymbol{z}_2$. In contrast, Aakala et al. (2023) identified stronger associations between population density and conifer dominance. The difference may stem from the capacity of our model to isolate spatial scales, revealing that such associations, though apparent in aggregated models, do not persist across spatial scales.

Regarding grazing impacts, on $\boldsymbol{z}_1$ and $\boldsymbol{z}_2$, BAs of both conifer tree species are negatively affected by grazing. Grazing can severely harm spruce, which naturally regenerates under deciduous canopies and gradually take over site dominance, but seedlings are often trampled in grazed forests. Birch BAs are for $\boldsymbol{z}_1$ positively associated, but negatively on $\boldsymbol{z}_2$. In comparison, BAs of other deciduous trees show a substantial positive effect. Deciduous trees have a pioneer strategy, in that they regenerate quickly on disturbed sites. However, cattle were often let into forest stands for grazing, which was in the recovery phase of slash-and-burn cultivation, and their grazing preferences further shaped the species composition. In particular, this led to an increase in alder that is poorly palatable for the cattle. Our results concerning grazing impacts are consistent with the findings of Aakala et al. (2023), where historical land-use intensity, including grazing pressure, is shown to have long-term effects on forest structure. Our spatial decomposition confirms these effects are more pronounced at the regional spatial scale.

Comparing the top and bottom panels of Fig. 6, it is evident that the respective $\boldsymbol{z}_1$ shows plot-to-plot variation in BA for each tree species studied. The respective estimated effective ranges on $\boldsymbol{z}_1$ are all smaller than the constructed distances between sample plots of 2 km. The estimated nugget effects ($\tau ^2$) are substantial for most of the estimated partial sill, so that there is still considerable location-independent variation in these details. The smoothing parameters (ν) are either estimated to be less than 0.5, or the effective range estimated zero. These results indicate for all tree species that there is little to no spatial correlation on $\boldsymbol{z}_1$. We detect scale-dependent dominant features on $\boldsymbol{z}_2$, which have effective ranges of approximately 40 km. These show no nugget effect on $\boldsymbol{z}_2$, and the smoothing estimates imply that close locations have similar values; that is, there are potentially connected patterns on these details. This means that we can identify a regional scale.

When we compare the spatial effects between the different tree species in Fig. 6, it is apparent that the estimates form four clusters, reflecting some differences between the respective spatial-dependency characteristics. The effective ranges (ρ) are similar, but the variance ($\sigma ^2$) and smoothness (ν) parameters differ. This is well reflected in the respective scale-dependent features shown in Figs. 4 and 9, 10 and 11 in the Supplementary Material, with equally sized large features. The features of other deciduous trees BAs are, however, less smooth than the other features.

Overall, cross-validation estimates in Fig. 5 show that the linear effects are stable. The cross-validation estimates of the spatial effects in Fig. 6 show that at most some regional artifacts affect the partial sill. However, the effective range and smoothness estimates are evidently stable and overall we do not detect any sign of overfitting. We also calculated the RMSE and CRPS for each training set, predicting to $\boldsymbol{x}$ from the sum of training $\boldsymbol{z}_1$ and $\boldsymbol{z}_2$. Table 1 in the Supplementary Material shows that these values are close across training sets, supporting that overfitting is not present.

## Discussion

Our application to Finnish forest inventory data demonstrates both the methodological success of extending scale-space analysis to geostatistical data and its substantive value for understanding ecological processes. Extending the dominant-feature identification for geostatistical data fills a major gap in spatial statistics and enables its application to a vast area of data. To make this method available for other researchers, the R functions and scripts are available in the git repository described in the Supplementary Material. The outlined method relies on the ML estimates of the Matérn covariance parameter, limiting the method to moderate data sizes. However, many approximations (e.g., covariance tapering or Vecchia approximations) are provided in spatial statistics, which can be plugged in at the necessary steps to overcome this limitation, see also Flury and Furrer (2022). Extending the feature attributes of scale-dependent features to additional linear effects offers further insight into possible drivers of the underlying processes. This novel aspect of the dominant-feature identification enables the analysis of associations to predictor variables, which potentially differ for different spatial scales. Especially predictors with large scales are likely to contain hidden small-scale effects. This novelty opens up a new range of applications and also previous dominant-feature identification approaches can be adapted accordingly. While the current approach selects smoothing scales λ independently, an alternative would be to model the scales with other model components jointly. However, this would lead to a dependency between the smoothing scales and the fixed effects, which may be undesirable.

Turning to the broader implications of the application, our analysis revealed that there are two essential scales with interesting differences, the small plot-to-plot scale and the large regional scale. The results imply that features in BA data are driven by differences in site-type and anthropogenic variables on both scales. Similar results were found in Aakala et al. (2023) based on expert-selected scales. Hence, the approach presented here produces similar results but, importantly, reduces subjectivity associated with the selection of scales thus increasing both the reliability and the repeatability of the analysis. Obtaining the scales from the data also likely increases the chances of capturing the relevant scales for analyzing the factors potentially explaining the observed phenomenon. In general, the effect of site type is considerably stronger than slash-and-burn, which influenced large areas of Finnish landscapes in the past. The high plot-to-plot variation in the site type is typical for Fennoscandian boreal landscapes, which form a matrix of forests on varying edaphic conditions (i.e., bedrock, quaternary deposits) on mineral soils, open and forested peatlands, and lakes and other water bodies. Known phenomena such as the decline of spruce and the increase in other deciduous trees associated with slash-and-burn were in line with our expectations. We also found an increase in grazing areas common for other deciduous trees on both scales.

In conclusion, these scale-dependent models enable us to capture differently directed associations between predictors and BAs, for example, between slash-and-burn and birch, which show different behavior at small and large scales. This would be impossible to assess with conventional models. By extending dominant-feature identification to geostatistical data, we address a gap in spatial statistics and expand its applicability. The flexible, modular framework can be adapted to specific data and holds broad potential across disciplines such as public health, environmental science, agriculture, and socioeconomics-domains where processes often operate simultaneously at multiple scales.

## Figures and Tables

**Fig. 7:**
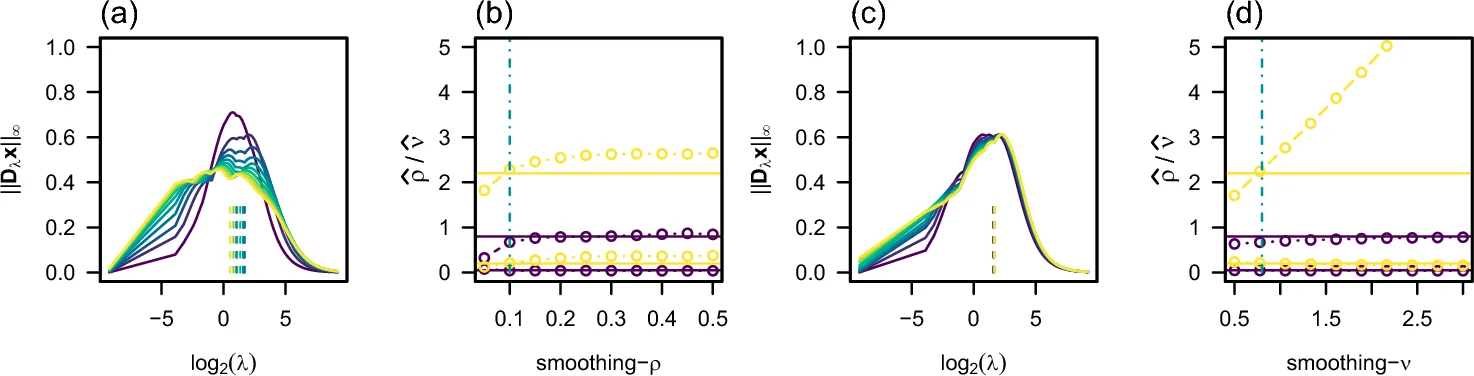
Smoothing correlation simulations: (a) and (c) scale derivatives for different effective ranges and smoothness parameters—the brighter the color of the scale derivative the higher the parameter. The vertical lines denote the respective local minima; (b) and (d) effective range and smoothness ML estimates of detail process $\boldsymbol{z}_1$ (dark color) and $\boldsymbol{z}_2$ (bright color) for different effective ranges/smoothness—solid horizontal lines depict the true parameters and dashed vertical lines show the optimal choices

**Fig. 8:**
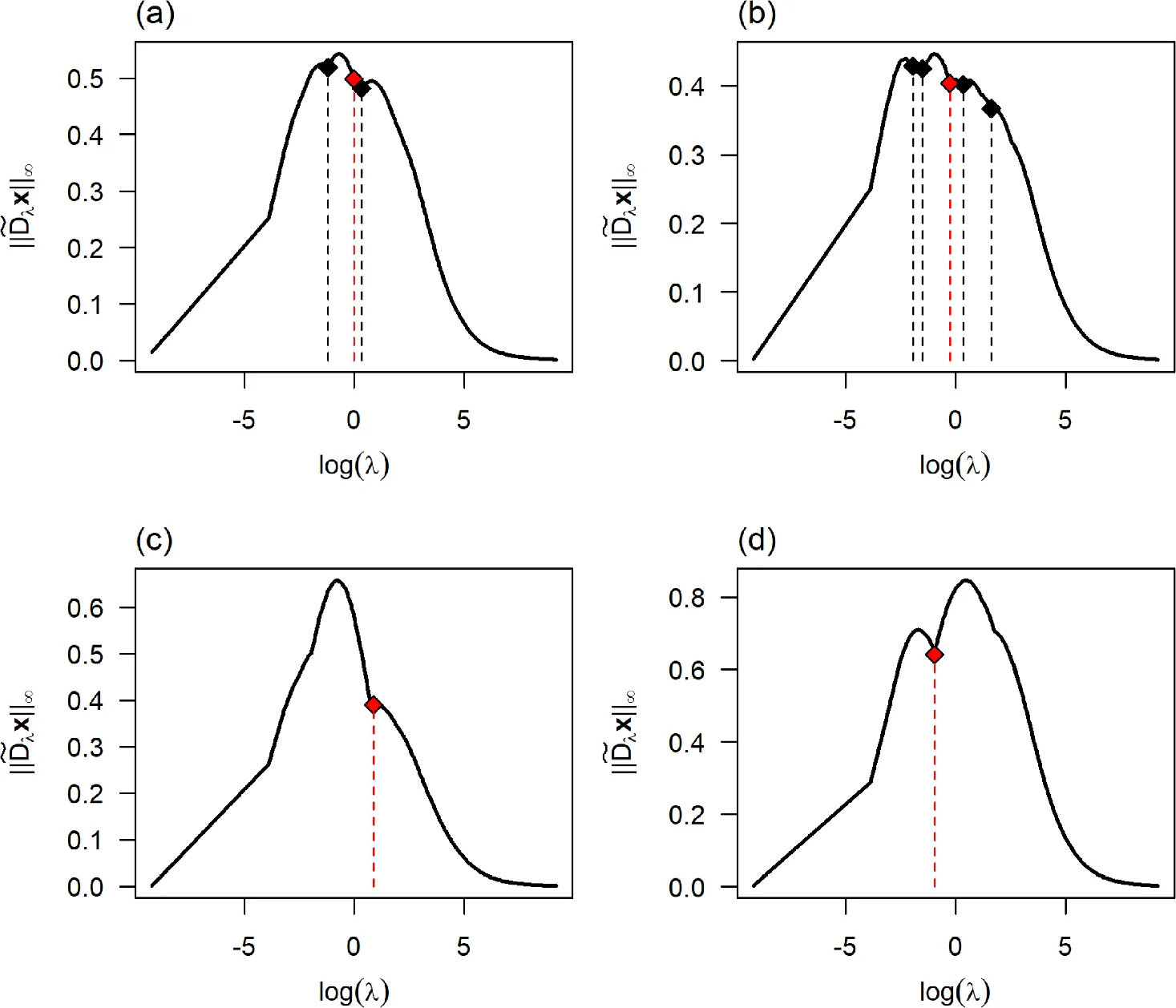
Scale-derivatives calculated across a sequence of smoothing parameters (λ). Diamonds indicate minima, with red diamonds marking the selected scales used for subsequent analysis. Panels show results for (a) pine, (b) spruce, (c) birch, and (d) other deciduous trees

## Data Availability

The NFI1 field data is held at the National Archives of Finland. The digitized NFI1 field data are property of the Natural Resources Finland (LUKE) and were used here with permission. Maps digitized in this project (population density and slash-and-burn agriculture) are available at: https://doi.org/10.6084/m9.figshare.23257562.

## Appendix A Source Files

Supplementary material is available in the git repository—https://git.math.uzh.ch/roflur/dominantfeaturesinfinnishforestdata information. It contains the following files:

- README.md: detailed description of available R-code.
- LICENSE: GNU general public license.
- source/: contains the R-devel package **mresa** to run the analysis.
- analysis/: contains R-scrips to run feature identification of Finnish forest inventory data.

## Appendix B Smoothing correlation Parameter

We use simulated data to illustrate how the tuning smoothing correlation parameters in $\boldsymbol{\widetilde{S}}_{\lambda }$ influence the decomposition and corresponding detail processes. We construct a composition of two fields by sampling from two zero-mean Gaussian processes, using different effective range and smoothing parameters for a Matérn covariance function ($\rho _1 = 0.05,\, \nu _1 = 0.8$ and $\rho _2 = 0.2,\, \nu _2 = 2.2$). The sampling domain for these processes is the unit square [0,1]×[0,1] with $2^{10}$ uniform randomly sampled locations.

We calculate the scale derivative for a sequence of effective range and smoothness parameters, choose the scale according to the local minima with respect to the norm of the maximum for each scale derivative and estimate the detail process parameters.

Panels (a) and (c) of Fig. 7 show the demeanor of the scale derivative. It is apparent that with increasing effective range and increasing smoothness parameters, the minima become stable at some point. Panel (b) and (d) of this figure visualize the same behavior for the parameter estimates of the detail processes; at some point also the effective range and smoothness stop changing, except for the smoothness of $\boldsymbol{z}_2$ process, which seems to increase with increasing smoothness parameter. Therefore, if the estimated smoothness parameter of detail $\boldsymbol{z}_{L-1}$ grows unreasonably large, this indicates that the smoothness parameter of the smoothing correlation function is chosen too large. These results show that the choice of the effective range and smoothness do not hamper the choice of the scale or of the detail process. However, for computational reasons, we propose to choose these parameters to be as small as possible.

## Appendix C Supplementary to Section “Applying dominant-feature identification to Finnish forest inventory data”

**Table 1:** Cross-validation prediction errors: RMSE and CRPS from predictions based on different training sets for each tree species

	RMSE				CRPS			
set/species	pine	spruce	birch	other	pine	spruce	birch	other
1	0.913	0.970	1.075	0.912	0.852	0.606	0.634	0.703
2	0.814	0.970	1.035	0.984	0.821	0.599	0.670	0.870
3	0.819	0.989	1.013	0.880	0.699	0.662	0.665	0.763
4	0.892	1.002	1.246	0.937	0.860	0.655	0.578	0.820
5	0.792	0.928	1.075	0.870	0.836	0.619	0.703	0.809
